# Prognostic and predictive value of supradiaphragmatic lymph node involvement detected by 18F-FDG PET/CT in advanced ovarian cancer: a systematic review and meta-analysis

**DOI:** 10.1007/s00404-025-08234-w

**Published:** 2025-10-29

**Authors:** Christian Braun, Julia Peikert, Christine Brambs

**Affiliations:** Department of Gynecologic Oncology, Lucerne Cantonal Hospital – Women’s Hospital, Spitalstrasse 16, 6000 Lucerne, Switzerland

**Keywords:** Advanced ovarian cancer, FDG-PET-positive supradiaphragmatic lymph nodes, 18F-FDG PET/CT, Cytoreductive surgery, Overall survival

## Abstract

**Objective:**

To evaluate the prognostic and predictive significance of supradiaphragmatic lymph node (SDLN) positivity detected by 18F-fluorodeoxyglucose positron emission tomography/computed tomography (18F-FDG PET/CT) in patients undergoing primary cytoreductive surgery for advanced epithelial ovarian cancer.

**Methods:**

A systematic review and meta-analysis was conducted according to PRISMA 2020 guidelines and registered with PROSPERO. Studies reporting on overall survival (OS), progression-free survival (PFS), and complete cytoreduction (R0) in patients with and without 18F-FDG PET/CT-detected SDLN metastases were identified through comprehensive database searches conducted on December 18, 2024. Data from five retrospective, single-center studies comprising a total of 605 patients were included in the quantitative synthesis. Meta-analyses were performed using a random-effects model.

**Results:**

SDLN positivity on 18F-FDG PET/CT was significantly associated with worse survival outcomes and lower resection rates. The pooled hazard ratio (HR) for OS was 1.60 (95% CI 1.19–2.25, *p* = 0.002) and for PFS 1.53 (95% CI 1.19–1.96; *p* = 0.0009), indicating poorer prognosis in SDLN-positive patients. The odds of achieving complete cytoreduction were significantly reduced in this group (OR = 0.32, 95% CI 0.15–0.68, *p* = 0.003). While heterogeneity was low for progression-free and overall survival (*I*^2^ = 0%), moderate heterogeneity was observed in the analysis of complete cytoreduction (*I*^2^ = 54%). None of the included studies provided histologic confirmation of 18F-FDG PET/CT-positive SDLNs.

**Conclusions:**

18F-FDG PET/CT-detected SDLN positivity is associated with worse survival and lower resectability in advanced ovarian cancer. Due to lacking histologic confirmation and retrospective data, prospective validation is needed.

## Introduction

Ovarian cancer remains the leading cause of death from gynecologic malignancies in the Western world, with approximately 70% of cases diagnosed at an advanced stage [1, 2]. The standard of care for advanced disease is primary cytoreductive surgery (PCS) aiming for complete resection of macroscopic disease, followed by platinum-based chemotherapy [3, 4]. Accurate preoperative staging is essential to determine the feasibility of PCS and to guide treatment decisions between upfront surgery and neoadjuvant chemotherapy (NACT) followed by interval debulking [3].

Contrast-enhanced computed tomography (CT) of the chest, abdomen, and pelvis is routinely used for staging [5]. However, 18F-FDG PET/CT has increasingly been implemented to improve the detection of extra-abdominal disease and may influence surgical planning.

Supradiaphragmatic lymph nodes (SDLNs) were defined as paracardiac, cardiophrenic, anterior mediastinal, mediastinal (medial and posterior), supraclavicular, and axillary lymph nodes [6].

Several studies have reported that 18F-FDG PET/CT can identify SDLNs, often leading to upstaging to FIGO stage IV [6–9]. While PET-positive SDLNs are often interpreted as a sign of distant metastasis, their true prognostic significance remains unclear [10].

Conventional CT-based identification of enlarged SDLNs has previously been associated with poorer OS, higher tumor burden, increased rates of non-complete cytoreduction, and diaphragmatic tumor spread [11–15]. However, studies evaluating the impact of PET-positive SDLNs on surgical and survival outcomes have failed to consistently demonstrate a significant prognostic value, raising doubts about the clinical relevance of such findings [7, 10]. The low rate of histological confirmation via fine-needle aspiration of PET-positive supradiaphragmatic nodes raises the suspicion that many of these nodes may be merely reactive—e.g., due to upper respiratory tract infections—and could therefore lead to false upstaging [16].

Given these conflicting data, the role of PET-positive SDLNs in clinical decision-making remains uncertain. In clinical practice, their presence may lead to the deferral of primary debulking despite a lack of robust prognostic evidence. This systematic review and meta-analysis aims to synthesize current evidence on the association of PET-positive SDLNs with OS, PFS, and R0 in patients undergoing primary debulking for advanced ovarian cancer.

## Methods

This systematic review was conducted according to the Preferred Reporting Items for Systematic Reviews and Meta-Analyses (PRISMA) 2020 guidelines. The protocol was prospectively registered in PROSPERO (CRD42024628681) on December 17, 2024. The aim was to assess the impact of supradiaphragmatic lymph node metastases detected by PET/CT on survival and clinical outcomes in epithelial ovarian cancer patients. Eligible studies included clinical trials and randomized controlled trials involving patients with epithelial ovarian cancer undergoing FDG PET/CT imaging for evaluation of supradiaphragmatic lymph node involvement at primary staging. Studies comparing patients with and without FDG PET/CT-detected supradiaphragmatic lymph node metastases were included. Primary outcomes were overall survival (OS), progression-free survival (PFS), and residual disease.

### Search strategy

Electronic databases including PubMed, Web of Science, Cochrane Library, and Google Scholar were systematically searched on December 18, 2024. Search criteria included: (“Carcinoma, Ovarian Epithelial”[MeSH] OR “Ovarian Neoplasms”[MeSH]) AND (“Positron Emission Tomography Computed Tomography”[MeSH] OR “Positron-Emission Tomography”[MeSH]) for PubMed; and (“ovarian carcinoma” OR “ovarian cancer”) AND (“PET-CT” OR “Positron Emission Tomography Computed Tomography”) for Web of Science and Cochrane Library. In addition to the database searches, the first 300 results from Google Scholar were screened using the defined search terms. This cutoff was based on methodological guidance suggesting that relevant studies are typically concentrated among the top-ranked entries. Exclusions comprised case studies, review articles, study protocols, and conference abstracts, with no restrictions on language or publication date. Duplicates were removed using EndNote. Screening and full-text reviews were independently conducted by two reviewers (CBR, JPE) using the Rayyan QCRI app, with disagreements resolved by a third reviewer (CHB). Data extraction was performed by two reviewers (CBR, JPE) using a standardized form. As all included studies were observational cohort studies, the risk of bias was assessed using the Newcastle–Ottawa Scale (NOS), which evaluates selection, comparability, and outcome domains.

### Data synthesis

Data synthesis was carried out using RevMan Web, with effect estimates presented as mean differences and 95% confidence intervals visualized in forest plots. A random-effects model was used to account for expected heterogeneity between studies. No subgroup analyses were performed.

#### Definition supradiaphragmatic lymph nodes

For the purpose of this review, we considered all cardiophrenic, mediastinal, parasternal, supraclavicular, and axillary lymph nodes as supradiaphragmatic lymph nodes (SDLNs). While some authors provided more detailed anatomical classifications (e.g., Lee et al. defined SDLNs as paracardiac, cardiophrenic, anterior mediastinal, mediastinal, supraclavicular, and axillary nodes [6], others referred more broadly to any lymph node located above the diaphragm with increased FDG uptake on PET/CT, most frequently cardiophrenic or anterior mediastinal nodes [9, 10, 17, 18]. None of the included studies reported histopathological confirmation of PET-positive SDLNs.

## Results

### Search results

A total of 1723 records were identified through database searches, including Cochrane Library (*n* = 35), PubMed (*n* = 673), Web of Science (*n* = 715), and Google Scholar (first 300 hits). After removal of duplicates, 1311 records remained and were screened for relevance. Of these, 1,266 were excluded based on title and abstract screening.

The remaining 45 full-text articles were assessed for eligibility. Forty articles were excluded for the following reasons: no data on supradiaphragmatic lymph nodes (*n* = 13), abstract only or insufficient data (*n* = 13), wrong outcome (*n* = 6), duplicate publication (*n* = 1), different research question (*n* = 1), and missing data despite author contact (*n* = 4). For these latter four, corresponding authors were contacted to obtain additional data; however, three were excluded due to reporting non-relevant outcomes (no data on SDLN, OS, PFS, or resection status) [7, 19, 20], and in one case, the author did not respond [21].

We contacted the corresponding author of the Danish cohort published by Risum et al. (2008–2012), which comprises three related publications assessing the prognostic value of PET/CT in advanced ovarian cancer. Unfortunately, the author was unable to provide subgroup-specific data on residual disease (R0 rates) or hazard ratios (HRs) for overall survival (OS) and progression-free survival (PFS) for patients with supradiaphragmatic lymph node metastases. As these outcomes were essential for our quantitative synthesis, we decided to exclude these three studies from the meta-analysis.

**Fig. 1:**
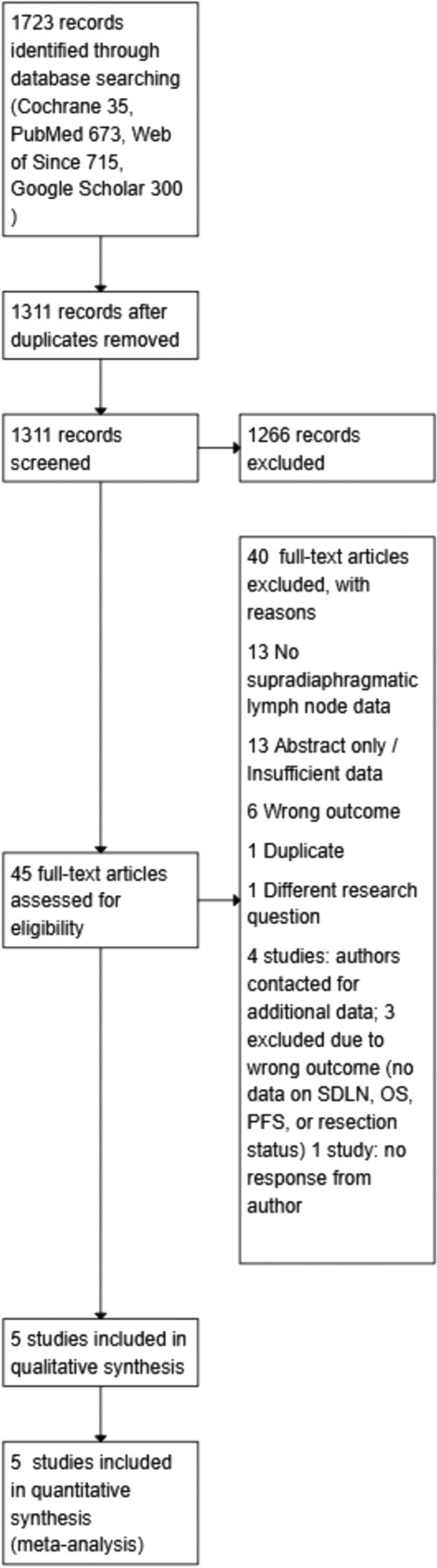
PRISMA flow diagram. Flow diagram illustrating the selection process of eligible studies for inclusion in the systematic review and meta-analysis, according to PRISMA 2020 guidelines

Finally, 5 studies were included in the qualitative synthesis, and all 5 were eligible for inclusion in the quantitative synthesis (meta-analysis) (Fig. 1).

### Characteristics of included studies

Sample sizes ranged from 53 to 176 patients, with a total of 605 patients included across all studies. The studies were conducted at single academic centers in Switzerland, France, Italy, and South Korea, reflecting geographic diversity but limiting generalizability due to the monocentric design.

Median progression-free survival (PFS) and overall survival (OS) were generally shorter in SDLN-positive patients, although in some studies (e.g., Braun et al., Park et al.), survival times were similar between groups and did not reach statistical significance, with the exception of Bats et al. (HR 5.70; 95% CI 1.74–18.6), which showed a statistically significant association between SDLN positivity and overall mortality.

The likelihood of achieving complete cytoreduction (R0) was consistently lower in SDLN-positive patients, with statistically significant differences observed in Lee et al.; other studies, such as Park et al., reported similar trends but without reaching significance. Most studies defined R0 as microscopic complete resection, whereas Fruscio et al. considered residual tumor of less than 1 cm as complete cytoreduction.

Except for Bats et al., all patients underwent primary debulking surgery (PDS). For the R0 resection analysis, only those patients from the Bats study who had undergone PDS were included in the meta-analysis.

Notably, none of the included studies performed histological confirmation of supradiaphragmatic lymph node involvement; PET/CT findings were used exclusively for SDLN classification.

None of the included studies systematically reported the macroscopic CT characteristics of PET-positive SDLNs or stratified nodes according to PET/CT concordance. Consequently, it remains unclear to what extent PET-positive SDLNs were also detectable by CT criteria alone, and whether PET provided incremental information beyond CT. Details on median follow-up duration and follow-up protocols can be found in Table 1.

**Table 1:** Characteristics and survival outcomes of included studies

Study	*n*	SDLN⁺/SDLN⁻ (*n*)	Histologic confirmation	PFS	OS	PDS/IDS (*n*)	R0 rate (SDLN-vs. +)	Median follow-up	Center/country
Bats et al. 2012	53	17/36	No	SDLN⁺ = HR 1.16, CI 0.72–3.96	SDLN⁺ = HR 5.7, CI 1.74–18.6	16/37	80.6% vs. 35.3%	17 mo	Hôpital Européen Georges-Pompidou, Paris/France
Braun et al. 2024	53	18/35	No	No PFS data	SDLN⁻ 60.76 mo	53/0	82% vs. 66.7%	70.1 mo	Luzerner Kantonsspital/Switzerland
					SDLN⁺ 58.76 mo				
Fruscio et al. 2013	95	25/70	No	SDLN⁻ 17 mo	SDLN⁻ 51 mo	95/0	53% vs. 20%	30 mo	San Gerardo Hospital, Monza/Italy
				SDLN⁺ 17 mo	SDLN⁺ 41 mo				
Lee et al. 2018	295	40/108	No	SDLN⁻ 18 mo	SDLN⁻ 37.5 mo	176/119	26.1% vs 27.8%	not specified	Yonsei University, Seoul/S. Korea
				SDLN⁺ 14 mo	SDLN⁺ 31.5 mo				
Park et al. 2024	109	36/73	No	SDLN⁺ = HR 1.62, CI 0.98–2.66	SDLN⁻ 45.73 mo	109/0	54.8% vs. 32.4%	28.1 mo	Kyung Hee Univ. Hosp., Goyang and Seoul/S. Korea
					SDLN⁺ 46.5 mo				

This table summarizes the key characteristics and survival outcomes of studies included in the meta-analysis, including study size, supradiaphragmatic lymph node status (SDLN⁺/⁻), histological confirmation, progression-free survival (PFS), overall survival (OS), type of surgical approach (PDS vs. IDS), complete resection (R0) rates, follow-up time, and study origin

### Study quality assessment

All five studies were retrospective and showed a generally moderate risk of bias, as expected in retrospective designs. All studies provided complete outcome data and reported their results transparently. Overall, study quality was moderate, but consistent outcomes and low heterogeneity support the validity of the findings. Details of the NOS assessment are provided in Table 2.

**Table 2:** Quality assessment of included studies using the Newcastle–Ottawa Scale (NOS)

Study	Representativeness exposed cohort	Selection non-exposed cohort	Ascertainment of exposure	Demonstration that outcome was not present at start	Comparability of cohorts	Assessment of outcome	Was follow-up long enough for outcomes to occur?	Adequacy of follow-up of cohorts	Total NOS-Score
Bats et al. 2012	★	★	★	★	★	★	–	–	6
Braun et al. 2024	★	★	★	★	★	★	★	★	8
Fruscio et al. 2013	★	★	★	★	★	★	★	–	7
Lee et al. 2018	★	★	★	★	★	★	★	★	7
Park et al. 2024	★	★	★	★	★	★	★	★	7

This table presents the methodological quality ratings of the included observational studies based on the Newcastle–Ottawa Scale, evaluating selection of participants, comparability of cohorts, and adequacy of outcome assessment

Due to the small number of included studies (*n* = 5), formal assessment of publication bias (e.g., funnel plot or Egger’s test) was not performed, as such methods are considered unreliable with fewer than 10 studies. Nevertheless, all included studies were retrospective, and the risk of selective reporting or non-publication of negative findings cannot be excluded.

This potential publication bias should be considered when interpreting the pooled effect estimates.

Given the limited number of included studies (*n* = 5), no meta-regression was performed, in line with methodological guidance discouraging its use in small meta-analyses.

A formal GRADE assessment was performed for the three meta-analyzed outcomes (OS, PFS, and R0 resection). Due to the retrospective nature of all included studies, the lack of histological confirmation of PET-positive SDLNs, and moderate imprecision in the pooled estimates, the overall certainty of evidence was rated as low across all outcomes (Table 3).

**Table 3:** GRADE summary of evidence certainty

Outcome	No. of studies	Study design	Risk of bias	Inconsistency	Indirectness	Imprecision	Publication bias	Certainty	Notes
Overall survival (OS)	5	Retrospective	Moderate	No concerns (*I*^2^ = 0%)	No concerns	Some concerns (CI 1.19–2.25)	Possible	⬤⬤◯◯ (Low)	Consistent effect, but retrospective design and lack of histologic confirmation lower certainty
Progression-free survival (PFS)	4	Retrospective	Moderate	No concerns (*I*^2^ = 0%)	No concerns	Some concerns (CI 1.19–1.96)	Possible	⬤⬤◯◯ (Low)	Similar limitations as for OS
Complete cytoreduction (R0)	5	Retrospective	Moderate	Moderate (*I*^2^ = 54%)	Some concerns (surrogate for surgical outcome)	Some concerns (wide CI: OR 0.15–0.68)	Possible	⬤⬤◯◯ (Low)	Similar limitations as for OS

Summary of the certainty of evidence for the three meta-analyzed outcomes (OS, PFS, and R0 resection), based on the GRADE approach. All outcomes were rated as low certainty due to study design limitations, imprecision, and lack of histological confirmation

### Meta-analysis results

The pooled analysis of overall survival (OS) across 5 studies showed a statistically significant prognostic impact of PET/CT-positive supradiaphragmatic lymph nodes. The combined hazard ratio (HR) was 1.60 (95% CI 1.19–2.25, *p* = 0.002), indicating that patients without PET/CT-positive SDLNs had a 37% lower risk of death. There was no evidence of statistical heterogeneity (*I*^2^ = 0%).

For progression-free survival (PFS), based on 4 studies, the pooled HR was 1.53 (95% CI 1.19–1.96; *p* = 0.0009), again favoring patients without SDLN involvement. Heterogeneity was minimal (*I*^2^ = 0%).

**Fig. 2:**
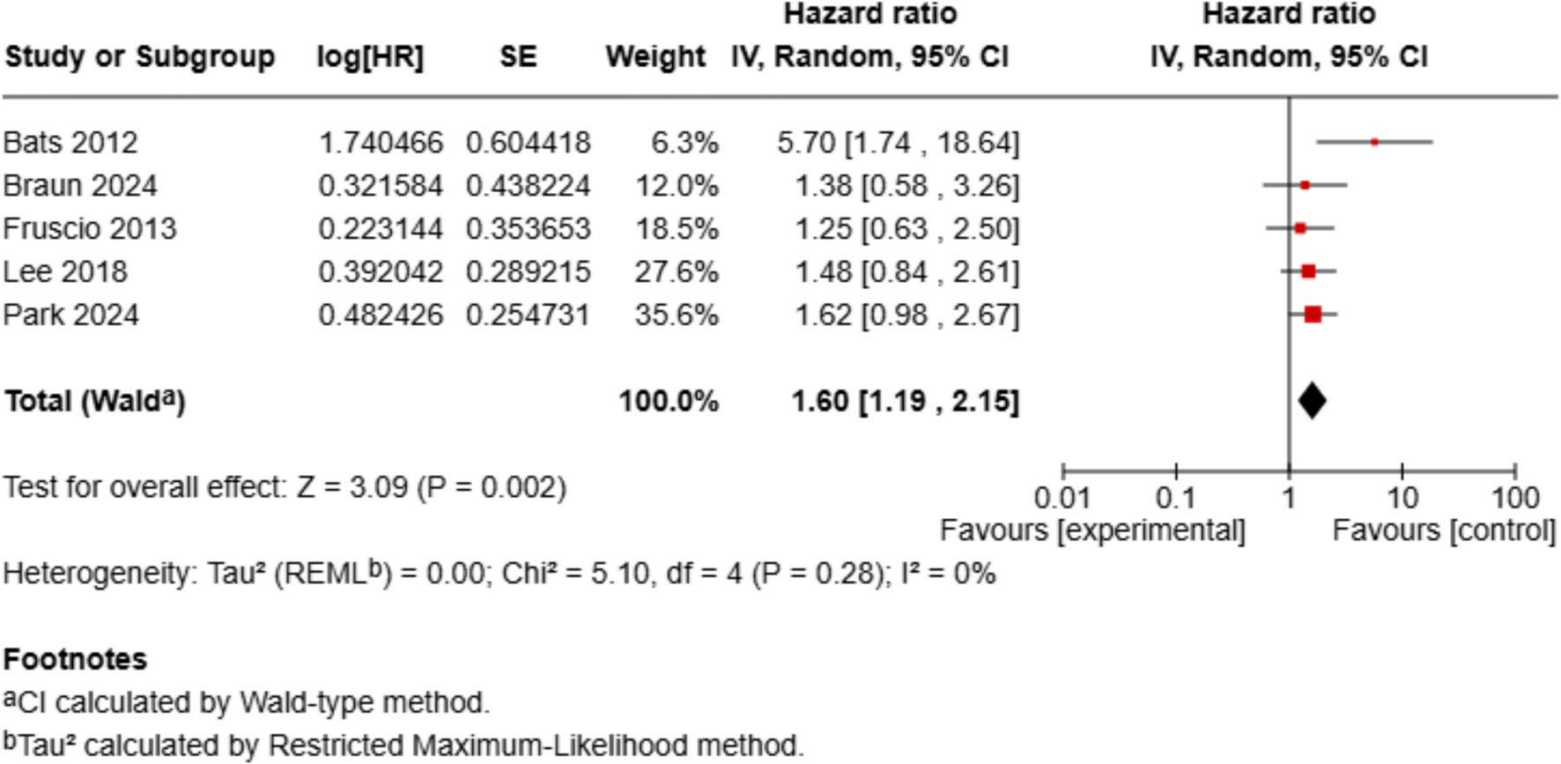
Forest plot of overall survival (OS). Forest plot displaying the hazard ratios (HRs) and 95% confidence intervals (CIs) for overall survival, comparing patients with and without supradiaphragmatic lymph node metastases (SDLN⁺ vs. SDLN⁻)

**Fig. 3:**
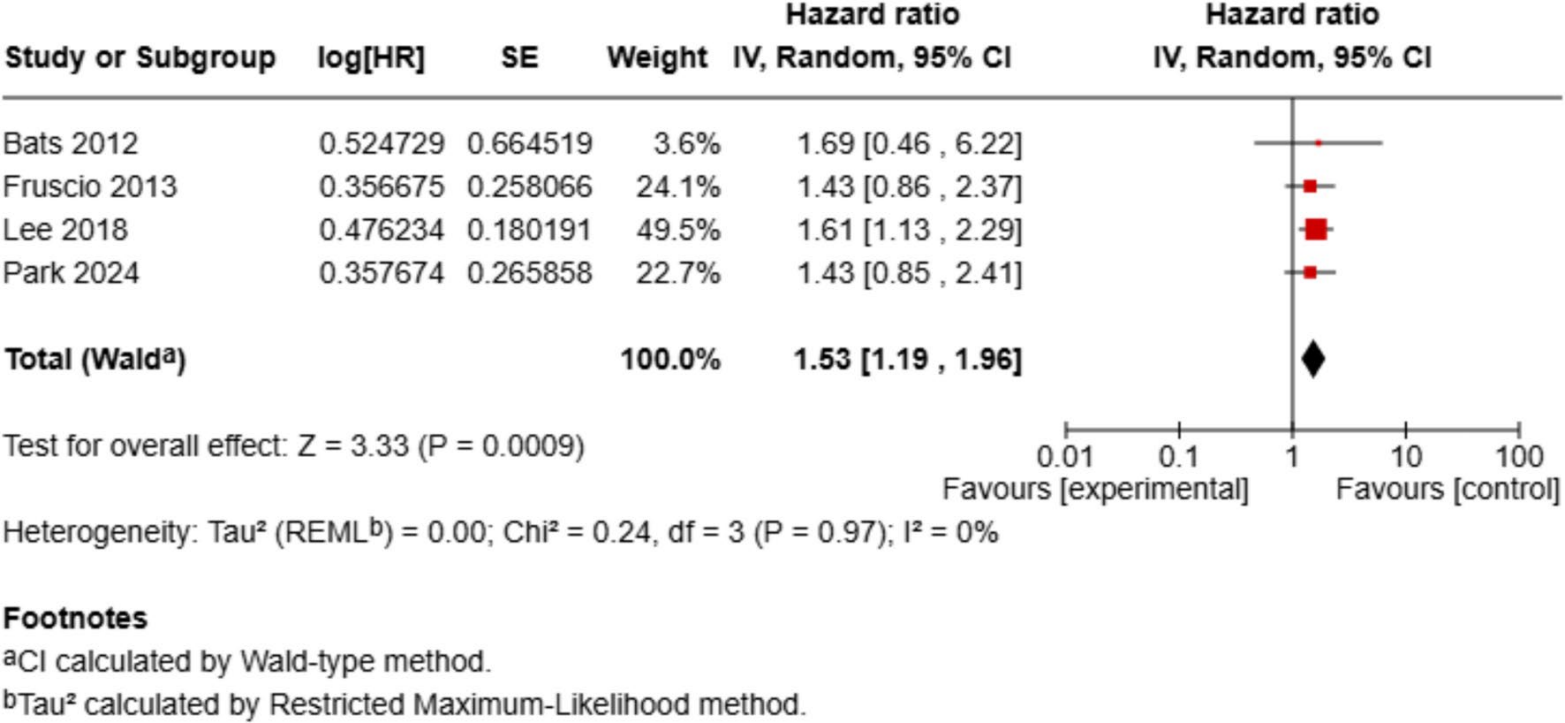
Forest plot of progression-free survival (PFS). Forest plot showing hazard ratios (HRs) and 95% confidence intervals (CIs) for progression-free survival, comparing SDLN⁺ and SDLN⁻ patient groups

**Fig. 4:**
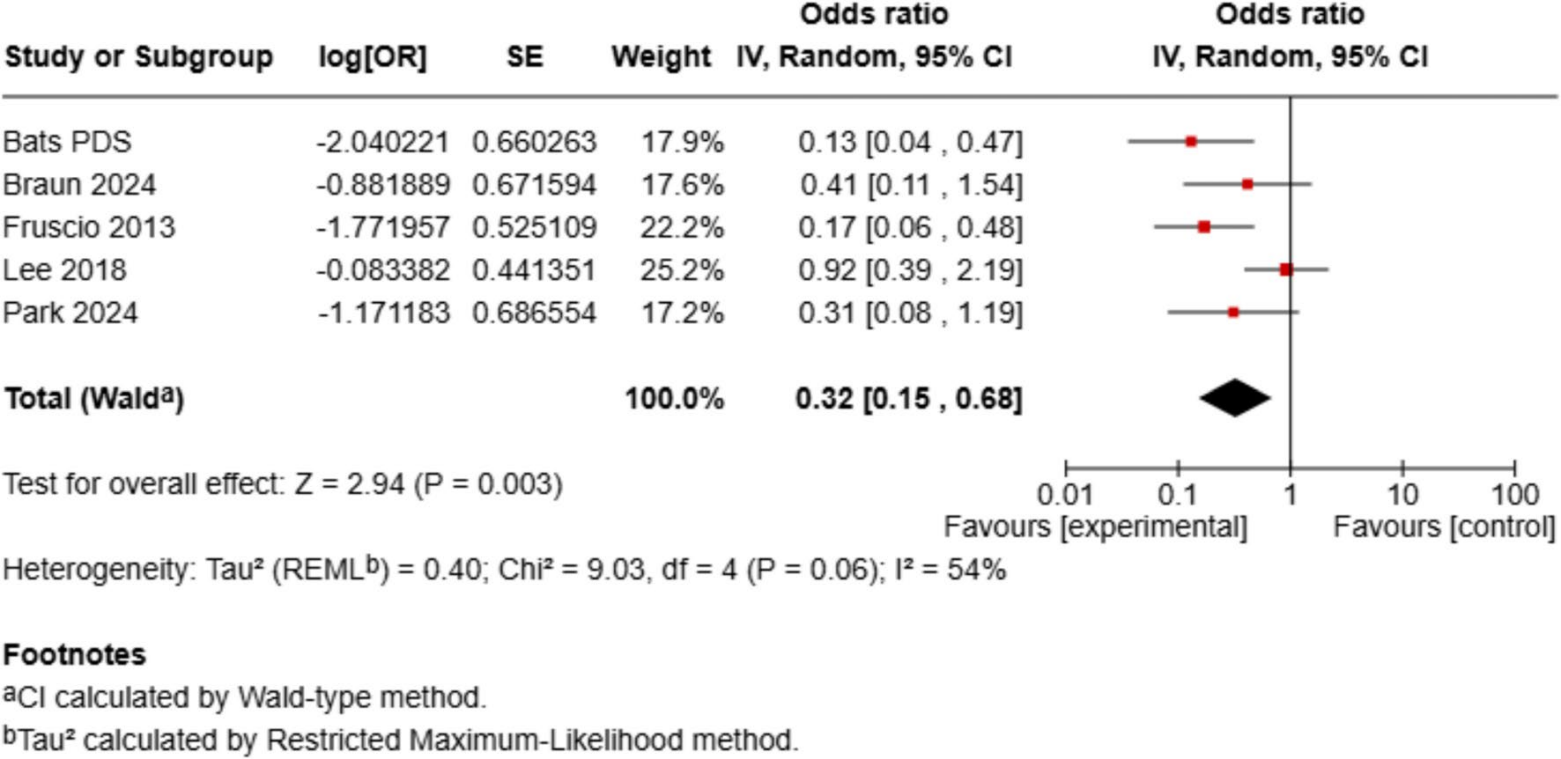
Forest plot of predicted surgical success (R0 resection rates). Forest plot presenting odds ratios (ORs) for complete macroscopic tumor resection (R0) during cytoreductive surgery in patients with SDLN⁺ versus SDLN⁻ findings on preoperative PET/CT

Regarding complete cytoreduction (R0 resection), 5 studies were included, and the presence of PET/CT-positive supradiaphragmatic lymph nodes was associated with significantly reduced odds of achieving complete cytoreduction (OR = 0.32, 95% CI 0.15–0.68, *p* = 0.003), with moderate heterogeneity (*I*^2^ = 54%). Only patients who underwent primary debulking surgery were included in our analysis. In the study by Bats et al., both primary and interval debulking procedures were reported, but we extracted and analyzed only the subgroup of patients who underwent primary debulking surgery. In addition, Fruscio et al. did not distinguish between macroscopic complete resection (R0) and residual disease ≤ 1 cm, which may limit comparability (Figs. 2, 3 and 4).

## Discussion

This systematic review and meta-analysis investigated the prognostic and predictive value of PET/CT-positive supradiaphragmatic lymph nodes (SDLNs) in patients undergoing cytoreductive surgery for advanced epithelial ovarian cancer. SDLN positivity was associated with poorer survival outcomes and lower rates of complete cytoreduction (R0). While several studies reported lower R0 rates and trends toward poorer OS and PFS in SDLN-positive patients, a statistically significant association was only observed in the study by Bats et al. (HR 5.70; 95% CI 1.74–18.6) [6, 17]. The lack of statistical power in smaller studies and the absence of histological confirmation—raising the possibility of false positives—may have contributed to these inconsistencies [9, 10, 17, 18].

Despite these limitations, our pooled analysis of 605 patients is the first to demonstrate a consistent association between SDLN positivity and all three clinically relevant endpoints: OS, PFS, and R0 resection. Rather than representing reactive nodes due to unrelated conditions such as upper respiratory tract infections, SDLNs may reflect true metastatic spread or secondary immune activation in the context of extensive peritoneal disease, reinforcing their biological and clinical relevance [16].

Our findings therefore reflect the prognostic association of PET-detected SDLNs in general, without establishing whether PET-only nodal positivity carries independent prognostic value or whether CT alone would have sufficed to identify clinically relevant SDLNs.

In comparison to Prader et al.’s CT-based study on cardiophrenic lymph nodes (*n* = 350), which demonstrated a stronger association with PFS (HR 1.91; 95% CI 1.26–2.89) and a modest association with OS (HR 1.62; 95% CI 1.19–2.89), our PET/CT-based analysis revealed a comparable effect on OS (HR 1.60; 95% CI 1.19–2.15) but a weaker association with PFS. This difference may be partly attributed to the lack of histologic confirmation in PET/CT studies, which increases the risk of false-positive findings and may limit interpretability [14].

The therapeutic implications of SDLN involvement, however, remain unresolved [14]. While histologic verification may improve prognostic accuracy and help differentiate metastatic from reactive findings, the clinical benefit of resecting SDLNs is unclear [14]. In line with findings from the LION trial, surgical removal of non-regional lymph nodes, including SDLNs, has not demonstrated a survival advantage. This supports the interpretation that SDLN positivity reflects tumor burden or disease dissemination rather than a modifiable therapeutic target [22].

From an anatomical perspective, SDLN involvement has frequently been linked to upper abdominal and diaphragmatic peritoneal carcinomatosis in CT-based studies, suggesting these nodes may serve as markers of widespread peritoneal disease rather than isolated nodal metastasis [14]. Although this association has not been consistently reproduced in PET/CT cohorts, the discrepancy may reflect methodological differences or insufficient sample sizes [10]. Nevertheless, SDLN positivity could help identify patients who would benefit from referral to specialized centers for upper abdominal cytoreduction.

### Limitations

Our analysis has several limitations. All included studies were retrospective, increasing the risk of selection and reporting bias. None of the studies provided histological confirmation of SDLN status, limiting the ability to distinguish true from false positives. Definitions of PET-positive nodes varied across studies, and follow-up protocols were inconsistent. In addition, none of the studies systematically reported CT morphology of PET-positive SDLNs or stratified findings according to PET/CT concordance, which precludes assessment of whether PET-only nodal positivity constitutes an independent risk factor or whether standard CT alone would have been sufficient for detection. Most studies were small, single-center cohorts—some published before 2013—reducing their applicability to current imaging technologies and surgical approaches, and the limited number of available studies (five for OS, four for PFS) together with the lack of consistent statistical significance across individual cohorts further restricts the strength of our conclusions. Furthermore, important clinical and biological prognostic factors such as tumor stage, pattern of peritoneal spread, ascites, ECOG performance status, BRCA or HRD status, and the use of maintenance therapies were not reported. This lack of information limits the ability to determine whether PET-positive SDLNs are an independent prognostic factor or simply reflect an increased overall tumor burden. Another limitation is that all patients in the included studies underwent primary debulking surgery. Thus, our findings cannot be generalized to patients treated with neoadjuvant chemotherapy and interval debulking or to those undergoing secondary or tertiary cytoreductive procedures.

### Future directions

Prospective, multicenter studies comparing PET/CT with conventional CT—ideally with histological confirmation—are needed to clarify the prognostic value of SDLN involvement and evaluate whether PET/CT provides superior prognostic value for surgical outcomes compared to standard CT and validate its utility for surgical planning.

## Conclusion

SDLN positivity detected by PET/CT appears to hold prognostic and predictive value in advanced ovarian cancer; however, its clinical utility remains uncertain. The prognostic relevance of SDLN status should be interpreted with caution and always in the context of additional clinical, pathological, and molecular factors. Current evidence is limited by the lack of histological confirmation, and PET/CT findings alone are not yet sufficient to guide treatment decisions. Nevertheless, SDLN positivity may serve as a marker to identify patients who should be referred to specialized centers with high expertise in upper abdominal surgery. Further prospective, histologically validated studies are essential to confirm the role of SDLN status as a reliable biomarker for clinical decision-making and should directly compare PET/CT with conventional CT while accounting for key clinical, pathological, and molecular prognostic factors.

## Data Availability

No datasets were generated or analyzed during the current study.
